# Retraction: Multimodal targeting of tumor vasculature and cancer stem-like cells in sarcomas with VEGF-A inhibition, HIF-1α inhibition, and hypoxia-activated chemotherapy

**DOI:** 10.18632/oncotarget.28560

**Published:** 2024-02-08

**Authors:** Changhwan Yoon, Kevin K. Chang, Jun Ho Lee, William D. Tap, Charles P. Hart, M. Celeste Simon, Sam S. Yoon

**Affiliations:** ^1^Department of Surgery, Memorial Sloan Kettering Cancer Center, New York, NY, USA; ^2^Department of Medicine, Memorial Sloan Kettering Cancer Center, New York, NY, USA; ^3^Threshold Pharmaceuticals, South San Francisco, CA, USA; ^4^Abramson Family Cancer Research Institute, Perelman School of Medicine, University of Pennsylvania, Philadelphia, PA, USA

**This article has been retracted:** Oncotarget has completed its investigation of this paper. A concerned reader informed the journal of several instances of image duplication. Namely, there is internal duplication between two images in Figure 3C. In addition, two images in Figure 3C are duplicates of images in Figure 6F from a previously published paper [1]. There is also a second internal duplication of one image in both Figures 4B and 6B. In light of these issues, the journal requested clarification regarding these images from the authors. The provided explanations were deemed unacceptable by the Scientific Integrity Office. Based on these facts, Oncotarget has decided to retract this paper.

Original article: Oncotarget. 2016; 7:42844–42858. 42844-42858. https://doi.org/10.18632/oncotarget.10212
